# Percutaneous retrieval of an embolized left atrial appendage closure device from the left atrium in a patient with previous MitraClips

**DOI:** 10.1186/s12872-019-1170-8

**Published:** 2019-08-14

**Authors:** Manik Chopra, Yam-Hong Wong, Lars Søndergaard, Ole De Backer

**Affiliations:** 1 Heart Center, Rigshospitalet, Blegdamsvej 9, 2100 Copenhagen, Denmark; 2 Medicine and Geriatrics Department, Tuen Mun Hospital, 23 Tsing Chung Koon Road, Tuen Mun, Hong Kong

**Keywords:** Left atrial appendage closure, LAAC, Device embolization

## Abstract

**Background:**

Left atrial appendage closure is increasingly used. LAA closure procedures can rarely be complicated by device embolization, typically towards the aorta. In this case report, we describe the successful percutaneous retrieval of an embolized Amulet device from the left atrium in a patient with previous MitraClips.

**Case presentation:**

By guidance of intracardiac echocardiography (ICE) and under local anaesthesia, two guiding systems were introduced into the LA; one to stabilize the device against the lateral LA wall and one large system to retrieve the device. However, the LAA closure device was finally snared by a three-loop snare and pulled across the interatrial septum (IAS) without taking the device into a protective guiding sheath – and this without tearing the IAS.

**Conclusions:**

In conclusion, percutaneous retrieval of an embolized LAA closure device from the LA is feasible by transseptal approach under local anaesthesia and with ICE guidance.

**Electronic supplementary material:**

The online version of this article (10.1186/s12872-019-1170-8) contains supplementary material, which is available to authorized users.

## Background

Left atrial appendage (LAA) closure is increasingly recognized as a therapeutic option for patients with atrial fibrillation (AF) and absolute or relative contraindications to oral anticoagulation (OAC) [1–3]. Rarely, LAA closure procedures are complicated by device embolization. Percutaneus strategies for device retrieval have been successfully applied and described in earlier case reports, typically after embolization towards the aorta [4–6]. In this case report, we describe the successful percutaneous retrieval of an embolized Amulet device (Abbott, MN, USA) from the left atrium (LA) in a patient with previous MitraClips (Abbott, MN, USA).

## Case presentation

An 86 year-old male suffering from severe, symptomatic mitral regurgitation (MR) was referred to our center for percutaneous mitral valve repair by use of the MitraClip system – this procedure was performed successfully. As the patient was also known with permanent AF (CHADSVASc score of 3) and a history of recurrent bleeding from the rectum (following radiotherapy for prostate carcinoma), a percutaneous LAA closure was proposed to and accepted by the patient at discharge.

**Fig. 1:**
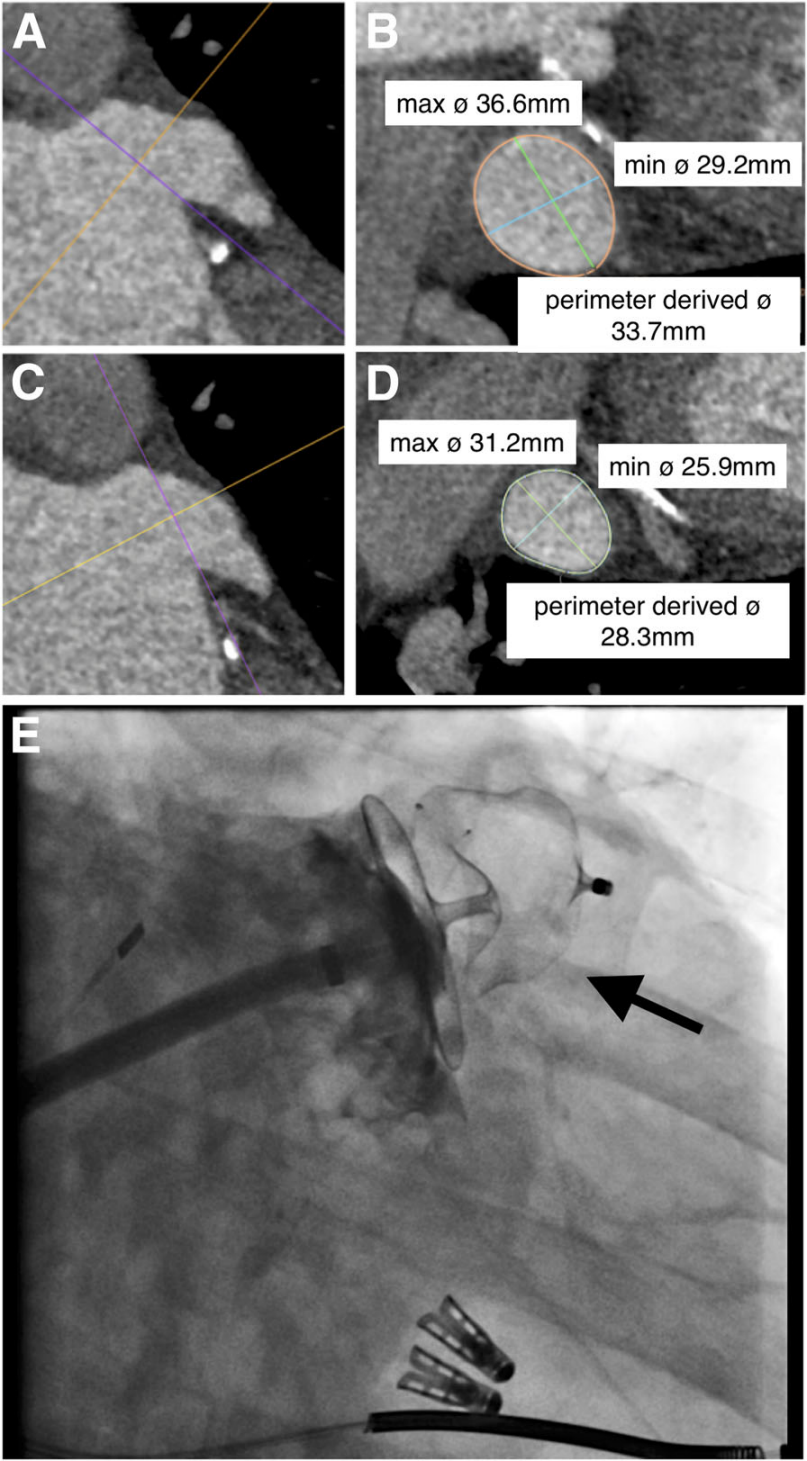
**a-b** CT measurements of the usual landing zone, and **c**-**d** CT measurements of a more distal alternative landing zone. **e** Amulet LA closure device released with signs of adequate stability and minimal leakage. CT, computed tomography

One month after MitraClip treatment, the patient was hospitalized for the percutaneous LAA closure procedure. Sizing of the LAA was performed by use of a pre-procedural multi-detector computed tomography (MDCT) scan, showing a LAA landing zone with a maximal diameter of 36.6 mm and a perimeter-derived mean diameter of 33.8 mm (Fig. 1a, b). This was at the upper range of LAA dimensions possible to close percutaneously, as the largest LAA closure device available is the Amulet device of 34 mm. Therefore, a particularly distal alternative landing zone was selected in an attempt to secure device anchoring. This second landing zone was measured to have a maximal diameter of 31.2 mm and a perimeter-derived mean diameter of 28.3 mm (Fig. 1c, d).

**Fig. 2:**
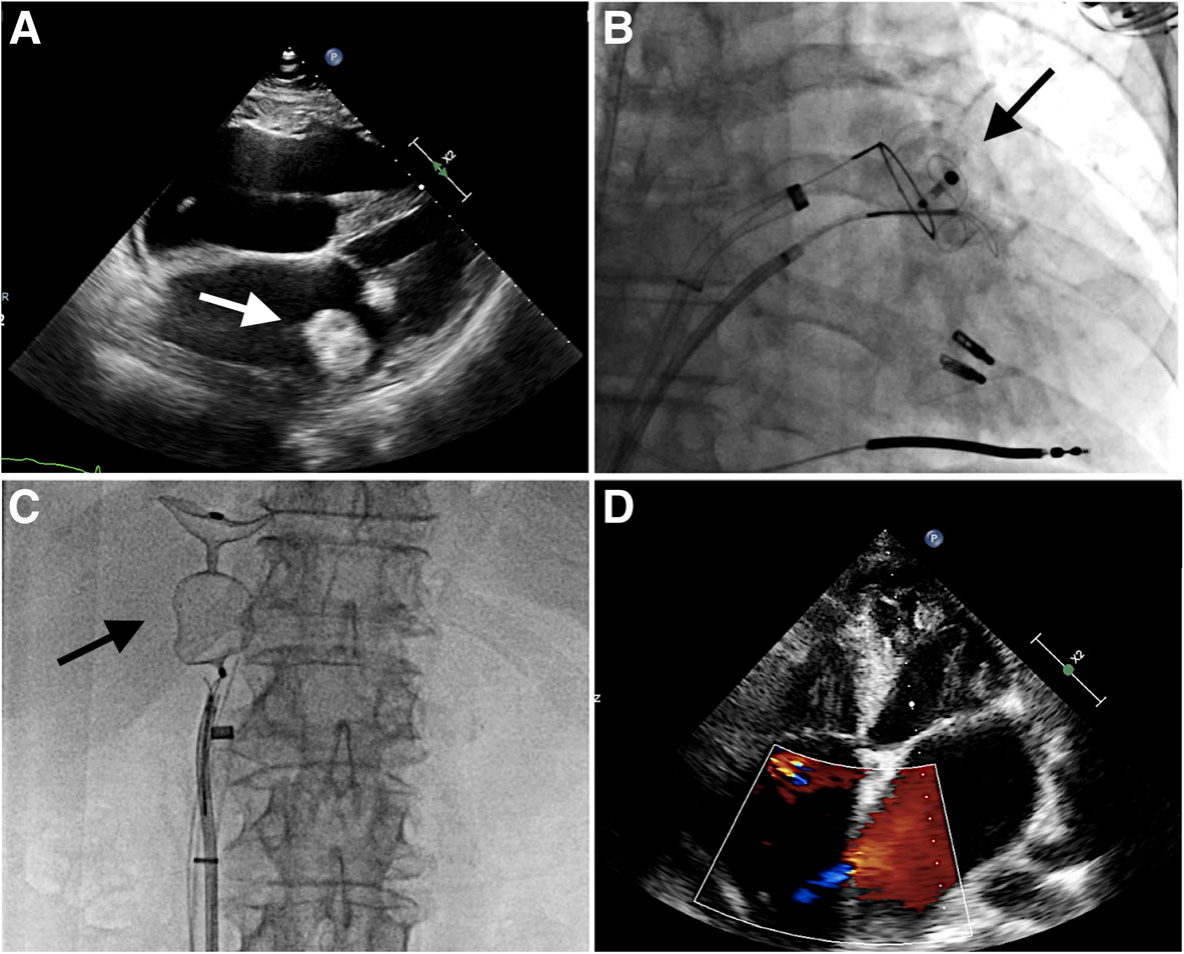
**a** Embolized Amulet device whirling in the left atrium. **b** Approach with two transseptal accesses for stabilization and retrieval of the embolized Amulet device, respectively. **c** Amulet device elongated and stabilized in the inferior vena cava before final retrieval into the MitraClip steerable guiding catheter and out of the patient. **d** Minimal shunt over the interatrial septum after retrieval of the embolized Amulet device


The LAA closure procedure was performed under local anesthesia and with guidance of intracardiac echocardiography (ICE). Given the LAA morphology and dimensions as described above, an Amulet device of 31 mm was implanted in this more distal position than usual in the LAA lobe. The lobe was implanted more than 2/3 beyond the circumflex artery, there was adequate compression on the lobe, the lobe was well-aligned with the axis of the LAA, there was adequate separation of the lobe and the disc, and the disc was concave-shaped. Also a gentle tug-test did not change the device position. Consequently, the LAA closure device was released (Fig. 1e). The patient remained stable during the post-procedural period; however, a follow-up transthoracic echocardiography (TTE) 18 h after the procedure showed device embolization (Additional file 1: Video S1). The cause of embolization was probably multifactorial: the trumpet-like large LAA with a wide ostium, and a short landing zone combined with a sharply angulated chicken-wing morphology. Since the patient had two MitraClips in place, the LAA closure device had no chance to migrate towards the aorta and the device was whirling around in the LA (Fig. 2a).

**Additional file 1: Video S1.** The emolized device whirling in the left atrium as seen on TTE. (MOV 11281 kb)


A strategy was made to retrieve the device percutaneously. The procedure was again performed under local anaesthesia under ICE guidance. One venous and one arterial access was made in the left groin, which were used for ICE and blood pressure monitoring, respectively. Two other venous accesses were taken in the right groin, in order to have two transseptal accesses into the LA. The plan was to stabilize the device with a forceps or snare through a steerable guide (the more inferior transsepal access) and ultimately snare and retrieve the device through a 24Fr MitraClip steerable guiding catheter (the more superior transseptal access) (Fig. 2b, Additional file 2: Video S2). In order to prevent aspiration of air through the MitraClip steerable guide, a short 16Fr sheath was introduced into the distal end of the MitraClip steerable guiding catheter.

A forceps and goose-neck snare were not successful to stabilize the wirling LAA closure device. Ultimately, the best and easiest strategy was to push the device against the lateral LA wall by means of a three-loop snare, introduced through the inferior transseptal access. As it turned out, by coincidence, that the screw on the distal end of the Amulet lobe presented itself in a very favorable way following this maneuver, the device was snared by means of the three-loop snare and retrieved from the LA by pulling the device through the transseptal puncture. As the steerable guide (Agilis NxT, Abbott, MN, USA) only had a 8.5Fr inner lumen, the Amulet device could not be pulled into this guide. Clearly, this entailed the (calculated risk) of inducing a major tear in the interatrial septum (IAS) - however, in such case, this could have been (most likely) been treated with an atrial septal defect closure device, which is readily available in our cathlab.

Following this maneuver, the device was released in the inferior vena cava (IVC). The MitraClip guiding catheter was then pulled back to the IVC (Fig. 2c) and the device was snared into the MitraClip system by use of a goose-neck snare. ICE showed an intact IAS and no pericardial effusion. All systems were taken out and the venous accesses were closed by figure-of-8 suture.

The patient remained hemodynamically stable throughout the entire procedure and heparin was given as per ACT (250–300 s) thoughout the procedure. The patient was stable during the post-procedural period. A control TTE 24 h after the procedure confirmed an intact IAS and no pericardial effusion (Fig. 2d). The patient was finally discharged on aspirin 36 h after the retrieval procedure.

## Discussion & Conclusion

When confronted with an embolized LAA closure device, percutaneous retrieval should be considered. Different techniques of embolized device retrieval have been well described [7], but an embolized device retained in the LA due to the presence of Mitraclips is not commonly encountered.

In case the LAA closure device migrates towards the aorta, snaring and retrieval by a retrograde approach using the femoral artery should be planned. In case the LAA closure device is caught in the LA, e.g. due to prior MitraClips, the device can be retrieved by transseptal approach. In the latter scenario, two systems could be introduced into the LA, one to stabilize the device against the lateral LA wall and one large enough system (≥ 16 Fr) to retrieve the device. However, in this specific case, the LAA closure device was pulled across the IAS without taking the device into a protective guiding sheath – this was done without damaging or tearing the IAS. The three-loop snare appeared to be the better choice for both stabilizing and retrieving the mobile LAA closure device and the best part of the Amulet device to snare is the screw on the distal end of the lobe.

In conclusion, percutaneous retrieval of an embolized LAA closure device from the LA is feasible by transseptal approach under local anaesthesia and with ICE guidance.

## Additional file

Additional file 2: **Video S2.** The emolized device whirling in the left atrium as seen on fluoroscopy, with a three-loop snare attempting to stablize the device. (MOV 34381 kb)

## Data Availability

Not applicable

## Abbreviations

AF: Atrial fibrillation
IAS: Interatrial septum
ICE: Intracardiac echocardiography
IVC: Inferior vena cava
LA: Left atrium
LAA: Left atrial appendage
MDCT: Multi-detector computed tomography
MR: Mitral regurgitation
OAC: Oral anticoagulation
TTE: Transthoracic echocardiography
